# Continuous Adaptive Evolution of a Fast-Growing *Corynebacterium glutamicum* Strain Independent of Protocatechuate

**DOI:** 10.3389/fmicb.2019.01648

**Published:** 2019-08-06

**Authors:** Michaela Graf, Thorsten Haas, Felix Müller, Anina Buchmann, Julia Harm-Bekbenbetova, Andreas Freund, Alexander Nieß, Marcus Persicke, Jörn Kalinowski, Bastian Blombach, Ralf Takors

**Affiliations:** ^1^Institute of Biochemical Engineering, University of Stuttgart, Stuttgart, Germany; ^2^Center for Biotechnology (CeBiTec), Bielefeld University, Bielefeld, Germany; ^3^Microbial Biotechnology, Campus Straubing for Biotechnology and Sustainability, Technical University of Munich, Straubing, Germany

**Keywords:** *Corynebacterium glutamicum*, adaptive laboratory evolution, continuous adaptive evolution, increased growth, protocatechuate, RamA, DtxR, RpoA

## Abstract

*Corynebacterium glutamicum* is a commonly applied host for the industrial production of amino acids. While valued for its robustness, it is somewhat inferior to competing strains such as *Escherichia coli* because of the relatively low growth rate of 0.40 h^−1^ in synthetic, industrial media. Accordingly, adaptive laboratory evolution (ALE) experiments were performed in continuous cultivation mode to select for a growth-improved host. To ensure industrial attractiveness, this ALE study aimed at a reduction of dependency on costly growth-boosting additives such as protocatechuate (PCA) or complex media supplements. Consequently, double selection pressures were installed consisting of a steady increase in growth rate demands and a parallel reduction of complex medium fractions. Selection yielded *C. glutamicum* EVO5 achieving 0.54 h^−1^ and 1.03 g_Glc_ g_CDW_^−1^ h^−1^ in minimal medium without abovementioned supplements. Sequencing revealed 10 prominent mutations, three of them in key regulator genes.

## Introduction and Motivation

Microbial bioprocesses play a major role in the production of biofuels, foods, food ingredients, feeds, cosmetics, and pharmaceuticals (Straathof, 2013). To ensure their economic competitiveness, processes are developed such that their performance is ideal under large-scale production conditions by either optimizing the producing biocatalysts, i.e., pro-/eukaryotic organisms or cellular parts (for example enzymes), or the surrounding process conditions. The latter comprises elements such as technical process setup, production strategy and corresponding process parameters, and the cultivation medium. Most often, the technical equipment is established in a production plant limiting optimization procedures to the process format and to the process parameters. Production media can be optimized regarding complexity and cost by utilization of simple salts as nitrogen-, phosphorus-, and sulfur sources and a cheap carbon source, e.g., molasses or corn steep liquor. Apart from extracellular process parameters, the production host can be improved as well. For this, metabolic engineering strategies, optionally combined with omics techniques (Becker and Wittmann, 2018), are applied to influence cell-specific attributes, e.g., growth rate, substrate spectrum, and uptake rate, or production rate, ultimately boosting productivity (Löffler et al., 2016; Nielsen and Keasling, 2016; Michalowski et al., 2017) or reducing cultivation media costs.

In contrast to targeted genome alterations applied in metabolic engineering, adaptive laboratory evolution (ALE) is used as a more traditional way to optimize the same attributes of a production organism (Dragosits and Mattanovich, 2013; LaCroix et al., 2015; Sandberg et al., 2016; Rugbjerg et al., 2018; Stella et al., 2019). In an ALE experiment, cells are exposed to specific selection pressures by creating clearly defined environmental conditions for a prolonged time period. Thereby, formation of evolved subpopulations is promoted and populations best adapted to the installed growth environment outperform the residual ones. This increase in fitness is caused by mutations in the genome of the evolved strain and is extrinsically observable by changed phenotypic characteristics, e.g., higher biomass-substrate yields, higher substrate consumption rates, or higher growth rates (Dettman et al., 2012; Ryall et al., 2012). Thus, ALE can be used in an industrial context to select for favorable mutations in a production host, e.g., robustness to (toxic) by-products (Tremblay et al., 2015; Mohamed et al., 2017; McCloskey et al., 2018), or adaption to non-native carbon sources (Lee and Palsson, 2010). In general, an ALE is performed in either batch or continuous process mode. While repetitive batch experiments are easy to conduct, environmental process conditions vary strongly. Besides decreasing nutrient supply and increasing population densities, pH and dissolved oxygen additionally fluctuate in non-controlled batch reactors such as shake flasks, potentially biasing the pursued outcome of the ALE. To prevent this, continuous bioreactors (e.g., chemostats) are employed to provide controlled process conditions, constant nutrient supply, and consequently constant population densities. Compared to repetitive batches, experimental efforts and costs of chemostats are potentially higher but justified considering that desired mutations are more likely to occur than in a batch ALE experiment (Dragosits and Mattanovich, 2013).

In this study, the Gram-positive microorganism *Corynebacterium glutamicum* was cultivated in a chemostat ALE experiment. This bacterium is considered an industrial workhorse of industrial biotechnology and has been engineered for production of numerous compounds, e.g., organic acids (Wieschalka et al., 2013), amino acids (Wendisch et al., 2016), bio-based fuels (Becker and Wittmann, 2012), diamines (Wendisch et al., 2018), heterologous proteins (Freudl, 2017), and grows on a wide range of substrates (summarized in Becker et al., 2016). Moreover, *C. glutamicum* shows physiological attributes that agree with large-scale production conditions: it is facultative anaerobic (Nishimura et al., 2007) and can endure fluctuating substrate concentrations, dissolved CO_2_ (Bäumchen et al., 2007; Blombach et al., 2013) and pH gradients (Follmann et al., 2009), and cannot be lysed by bacteriophages. Nevertheless, the most obvious drawback of this workhorse is the relatively small maximum growth rate (*μ*) of about 0.4 h^−1^ under conventional growth conditions using synthetic media, and of 0.6 h^−1^ installing specific process formats (Bäumchen et al., 2007; Grünberger et al., 2013) or growth media (Unthan et al., 2014; Graf et al., 2018). Considering that the overall process productivity is positively influenced by high growth rates for growth-coupled (Feist et al., 2009) and growth-decoupled cultivations, increasing *μ* would further boost the attractiveness of *C. glutamicum* in industrial applications. For this reason, two independent ALE experiments were recently performed with *C. glutamicum*, and indeed, the growth rate could be increased by employing repetitive batch ALEs (Pfeifer et al., 2017; Wang et al., 2018). But as pointed out above, the growth environment of the producing cell is another parameter that can be simultaneously targeted in an ALE to increase the overall process performance. In most (research-related) applications, *C. glutamicum* is cultivated in the standard mineral medium CGXII (Keilhauer et al., 1993; Kind et al., 2010) or MM (Liebl et al., 1989) with a carbon source of choice. Both media contain iron-chelators, i.e., protocatechuate (PCA, in CGXII) or catechol (in MM) that are believed to facilitate iron acquisition (Liebl et al., 1989) or may serve as additional carbon source fueling the citric acid cycle (Shen et al., 2012; Unthan et al., 2014). Aside from their unclear cellular function, especially PCA is a relatively costly medium component that is estimated to be 50–100 fold more expensive than the carbon source and should be prevented as additive to minimize media costs.

Consequently, this investigation had two aims: improving the growth performance of *C. glutamicum* and adapting the strain to an iron-chelator-free minimal medium. Thereby, both parameters should be optimized simultaneously to elevate cellular productivity that makes the organism an ideal choice for large-scale applications. To reach these goals, we employed a time-efficient serial continuous ALE concept and imposed increasingly challenging selection pressures on the strain. In that way, we especially aimed for the selection of mutations in regulatory genes that enhance the cell’s performance in the desired ways. Following this approach, we subsequently sequenced evolved strains to gain insights into genome mutations.

## Materials and Methods

### Genome Sequencing of *C. glutamicum* Strains ATCC 13032 and EVO5

For genome (re-)sequencing, DNA of *C. glutamicum* ATCC 13032 and the EVO5 strain was isolated by the NucleoSpin Microbial DNA Kit (Macherey-Nagel, Düren, Germany) according to the manufacturer’s instructions. The Illumina TruSeq PCR-free sample preparation Kit was used for library preparation, which was sequenced on a MiSeq system (Illumina, San Diego, CA, USA) by paired-end sequencing with a read-length of 2 × 300 bases. The sequencing reads were assembled by Newbler v2.8 (Roche, Branford, CT, USA) and genome finishing was done using the Consed software (Gordon, 2003). For detection of SNPs, the genome sequence of EVO5 was aligned to the reference genome ATCC 13032 by the software Snapgene v4.3 (GSL Biotech, available at snapgene.com), whereby SNPs were automatically detected.

### Bacterial Strains, Preculture, and Media

The wild-type strain *C. glutamicum* ATCC 13032 (WT) obtained from the American Type Culture Collection (ATCC, Manassas, VA, USA) was used in this study. *C. glutamicum* cells from a glycerol cell culture stock where spread on a 2× tryptone-yeast extract (YE) (2× TY, Sambrook, 2001) agar plate which was incubated at 30°C for 48 h. Subsequently, colonies extracted from the agar plate were used to inoculate 5 ml 2× TY medium in glass reaction tubes and incubated for 8 h at 30°C under constant agitation (120 rpm) on a bench-top rotary shaker (Infors HT, Bottmingen, Switzerland). Sterile 500 ml baffled shaking flasks were filled with 50 ml of modified minimal medium CGXII (Buchholz et al., 2014) supplemented with 4% (w/v) glucose. Individual pre-culture minimal medium flasks were inoculated with 5 ml from the pre-culture in glass reactions tubes and incubated overnight at 30°C and constant agitation (120 rpm).

### Bioreactor Batch Cultivations

Characterization experiments of strains *C. glutamicum* WT and EVO 5 were performed in a bioreactor (Bioengineering, Wald, Switzerland) with a volume of 3 L, equipped with a six-blade Rushton impeller, a dissolved oxygen (pO_2_)-, and pH-probe (Mettler Toledo GmbH, Albstadt, Germany). Off-gas analysis was enabled by non-dispersive (photometric) infrared gas analyzers (BCPO_2_ and BCPCO_2_, BlueSens, Herten, Germany) to determine molar O_2_ and CO_2_ fractions. Cultivations were conducted at 30°C and at a total pressure of 1.5 bar. pH was kept constant at 7.4 by titration with 25% (v/v) NH_4_OH. Abundance of oxygen was ensured by keeping the oxygen saturation above 30% through increase of the impeller speed in steps of 50 rpm and by stepwise increase of the aeration rate by 0.15 L min^−1^. The initial impeller speed was 250 rpm and the aeration rate 0.15 L min^−1^ (0.1 vvm). The amount of biomass needed to inoculate 1.5 L of media with an initial optical density (OD_600_) of 1 was harvested from the pre-culture shaking flasks, centrifuged at 4,000 × *g* (5430 R, Eppendorf, Hamburg, Germany) for 10 min and 4°C and resuspended in 100 ml sterile 0.9% (w/v) NaCl solution. The resulting biosuspension was used to inoculate 1.4 L CGXII minimal medium (Buchholz et al., 2014) supplemented with 2% (w/v) glucose. Antifoam agent (Struktol® J 647, Schill + Seilacher, Hamburg, Germany) was added manually when necessary. Batch cultivations were performed in independent biological triplicates.

### Continuous Evolution Experiments

#### Control Scheme of the Continuous Bioreactor

**Figure 1:**
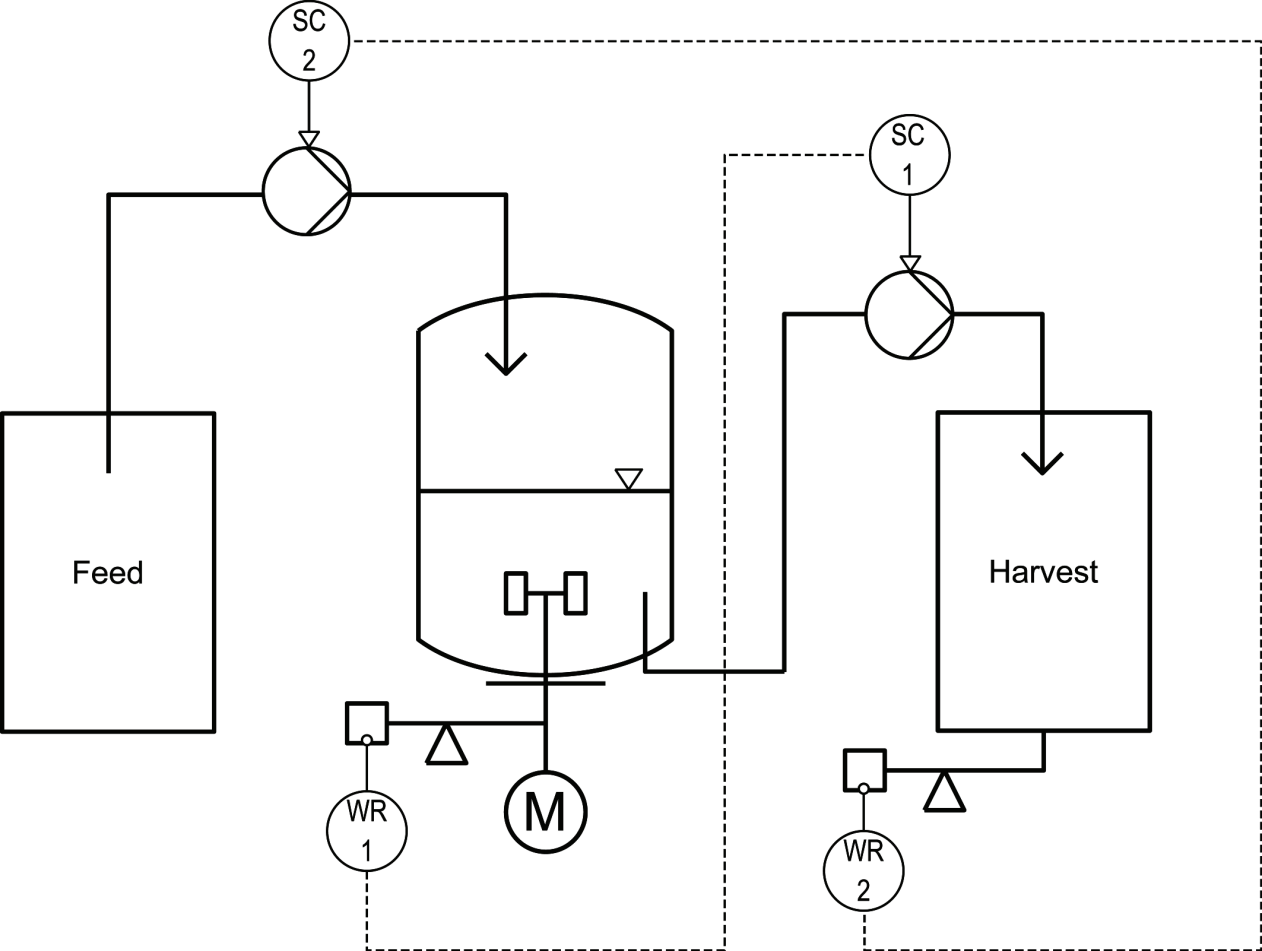
Control scheme of the continuous bioreactor for installment of near wash-out dilution rates. The first controller ensured an accurate reaction volume of 1.2 L by using the bioreactor weight (WR 1) as setpoint and adjusting the pumping rate of the harvest pump (SC 1) if the deviation was >0.2%. An accurate feed rate was automatically installed with the second controller that used the weight of the harvest (WR 2) and deduced mass flow as setpoint to adjust the speed of the feeding pump (SC 2) if the deviation was larger than 2%.

Continuous evolution experiments were performed with the same bioreactor setup as described in Section “Bioreactor Batch Cultivations.” During the continuous process mode, feed medium was constantly added from a reservoir with a peristaltic pump (Watson Marlow 120 U/DV 200 RPM, Falmouth, UK) and simultaneously, the same volume of the fermentation broth was harvested with a second pump. An accurate feed rate was automatically ensured by a controller (Figure 1) that continuously monitored the weight of the harvested material (laboratory scale Combics 3, Sartorius, Göttingen, Germany). It converted the measured mass flow into volume flow (density of the biosuspension: 1.01 kg L^−1^) and adjusted the influx by regulating the pumping speed when the deviation from the desired flux got larger than 2%. The reaction volume of 1.2 L was kept constant by monitoring the weight of the total reactor using a laboratory scale (Combics 3, Sartorius, Göttingen, Germany) and by automatically adjusting the speed of the harvest pump with a second controller which allowed deviation from the setpoint weight of 0.2%. This setup enabled setting dilution rates with a precision of 0.01 h^−1^. The regulation scheme was implemented in a custom process control system using the software LabVIEW (LabVIEW® 2010, National Instruments, Austin, TX, USA).

#### Execution of the Evolution Experiments

**Figure 2:**
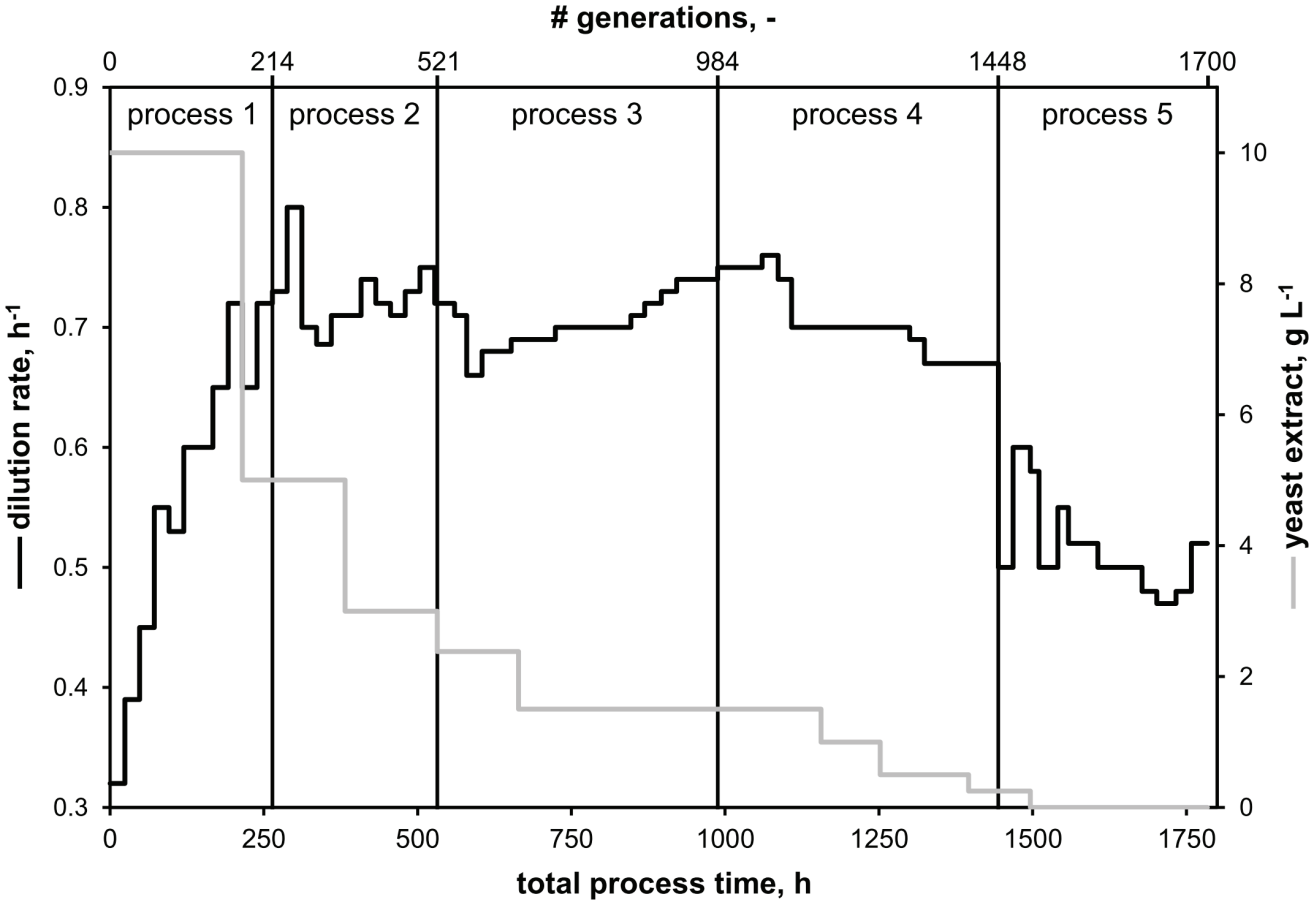
Overview of continuous evolution processes starting with *C. glutamicum* WT. Five processes were conducted using CGXII minimal medium supplemented with 1% (w/v) glucose and varying amounts of yeast extract (YE). The set dilution rate equaled the growth rate of the culture. After the first process, following processes were inoculated with potentially mutated strains from the respective previous process.

To start a continuous evolution bioprocess, sterile medium from the feed reservoir containing CGXII minimal medium (Buchholz et al., 2014) supplemented with 1% (w/v) glucose and varying amounts of YE was pumped into the sterile bioreactor until a volume of 1.1 L was reached. A batch cultivation was initiated using the same protocol as described in Section “Bioreactor Batch Cultivations” with a total reaction volume of 1.2 L and initial aeration rate of 0.12 L min^−1^. At the end of the batch phase, indicated by a sharp rise of the pO_2_ signal, the stirrer speed was set to 650 rpm and the aeration rate to 1.2 L^−1^ (1 vvm). Next, feed and harvest pumps were started and the pumping speed adjusted so that the primary dilution rate was equal to the exponential growth rate determined during the batch phase. About 60 μl h^−1^ of antifoam agent was added constantly with a syringe pump (LA-30, Landgraf HLL, Langenhagen, Germany). Whenever a steady state was reached, indicated by constant process parameters (pO_2_ and off-gas signals), the dilution rate was increased to approximate the maximum growth rate of *C. glutamicum* under current growth conditions. Additionally, the YE content of the feed medium was reduced stepwise as indicated in Figure 2. Each evolution experiment was performed for an average of 3 weeks. At the end of each experiment, samples of the presumably evolved strain were preserved in glycerol stocks and the most recent one was used to inoculate the following process.

### Re-engineering of a Mutation From Evolved Strain EVO5 Into *C. glutamicum* Wild Type

To re-engineer the genomic *ramA* mutation (C → G at nucleotide position 302 in the gene) in *C. glutamicum* ATCC 13032 wild type (WT) that was identified in evolved strain EVO5 (c.f., Section “Mutations in Evolved Strain EVO5 and Growth Characterization”) encoding RamA(S101C), the pK19*mobsacB* vector (Schäfer et al., 1994) was used. The 500-bp sequences flanking the point mutation were amplified with the primer pairs fw_ramA-flank1 + rev_ramA-flank1 (upstream region) and fw_ramA-flank2 + rev_ramA-flank2 (downstream region), respectively. The C → G mutation itself was encoded in the primer rev_ramA-flank1 and fw_ramA-flank2, respectively (Supplementary Table S1, marked in bold letters). Amplification products were inserted simultaneously in *Hin*dIII and *Bam*HI linearized pK19*mobsacB* by isothermal assembling in accordance with the protocol provided by Gibson (2011) yielding pK19*mobsacB*-*ramA*(S101C). Plasmids were amplified in *Escherichia coli* DH5α and purified using a commercially available kit (*E.Z.N.A. Plasmid Mini kit I*, Omega Bio-Tek Inc., Norcross, Georgia, USA). The insert was free of undesired mutations as verified by sequencing with primers fw_pK19-seq and rev_pK19-seq (GATC light run service). The point mutation was eventually introduced in the genomic background of *C. glutamicum* WT by a cassette exchange using pK19*mobsacB*-*ramA*(S101C) with the work-flow outlined by Schäfer et al. (1994). Electrocompetent cells of *C. glutamicum* ATCC 13032 were prepared according to the protocol provided by Tauch et al. (2002) with slight modifications: cultures were harvested at an OD_600_ of 1.75 and washed three times with 10% (v/v) glycerol. Transformation of competent *C. glutamicum* cells with 300–800 ng of purified plasmid by electroporation (Eppendorf Eporator, 2,500 V, 2 mm gap width, achieving time constants of 4.3–4.7 ms) was carried out following the optimized protocol by van der Rest et al. (1999). Strains carrying the correct *ramA* mutation were identified by sequencing. The mutated region was amplified with primers fw_ramA-flank1 and rev_ramA-flank2 and sequenced with primer fw_ramA-seq and rev_ramA-seq. Oligonucleotide sequences are provided in Supplementary Table S1.

### Analytical Methods

During batch cultivations in bioreactors, biosuspension was sampled hourly to determine the biomass concentration and the total inorganic carbon (TIC) species and to analyze substrate- and by-product concentrations in cell-free filtrates. Biomass formation was determined by measuring the OD_600_ (DR 3900, Dr. Lange, Berlin, Germany) and by preparation of cell-dry-weight (CDW) samples. For the latter, 1 ml biosuspension was washed twice in deionized water and centrifuged between each washing steps for 2 min at 20,173 × *g* and 4°C (5430 R, Eppendorf, Hamburg, Germany). The washed biomass was transferred to glass vials and dried for at least 24 h in a convection oven (Heraeus, Hanau, Germany) at 105°C. After cooling in a desiccator, vials were weighed on a micro-scale (AE 200, Mettler Toledo, Gießen, Germany). A correlation factor of 0.27 g L^−1^ for OD_600_-measurements to CDW was obtained. Cell-free samples were produced by filtering the biosuspension through 0.2 μm pore size syringe filters (Rotilabo®, Carl Roth, Karlsruhe, Germany). The filtrates were stored at −20°C until measurement. Glucose and pyruvate concentrations were determined with enzymatic assays following the manufacturer’s instructions (R-biopharma, Darmstadt, Germany). TIC amounts in biosuspension samples were determined as described by Buchholz et al. (2014) to correct for underestimated CO_2_ values gained by off-gas analysis during the batch cultivations. Biomass formation during shaking flask main cultivations was determined using hourly OD_600_ measurements. During evolution experiments, OD_600_ and cell-free samples were taken at least once per day.

### Determination of Kinetic Parameters

#### Growth Rate

The exponential growth rate (*μ*) in batch cultivations (shaking flask and bioreactor) was determined by linear regression of the logarithmic biomass concentration over the respective process time. During continuous bioprocesses, the set dilution rate (*D*) equals the growth rate since the feed rate and the bioreaction volume remained constant. This was ensured by the described controller scheme in Section “Control Scheme of the Continuous Bioreactor.”

#### Consumption Rates and Yields

The biomass-substrate yield (*Y*_XS_) was determined by linear regression of substrate and biomass concentration curves. The biomass-specific glucose consumption (*q*_Glc_) of *C. glutamicum* strains was calculated by dividing the determined exponential growth rate or the installed dilution rate (*D*) by *Y*_XS_.

#### Respiratory Rates

The biomass-specific respiratory rates (*q*O_2_,*q*CO_2_) were obtained by dividing the volumetric oxygen consumption or carbon dioxide emission rates by the biomass concentration. As previously described (see above), the carbon dioxide emission rate was then corrected by TIC-measurements using a total carbon (TC) analyzer (Multi N/C 2100s, Analytik Jena, Jena, Germany).

## Results

### Evolution Experiments

A total of five consecutive continuous bioreactor processes were conducted with the goal of increasing the growth rate of *C. glutamicum* with an evolutionary approach thereby selecting for the fastest-growing subpopulation. Minimal medium CGXII supplemented with 1% (w/v) glucose, omitting the standard CGXII component PCA (Keilhauer et al., 1993; Kind et al., 2010), and varying amounts of YE was used as growth medium. All processes are illustrated in Figure 2 with the total process time as sum of all cultivations. By analogy, total numbers of generations are calculated from the respective dilution rates and corresponding time periods. The first cultivation started with *C. glutamicum* WT and supplementation of 10 g YE L^−1^ in the feed medium. The initial dilution rate (*D*), mirroring the growth rate, was increased stepwise from 0.32 to 0.72 h^−1^ within 8 days (d, corresponds to 142 generations, black curve in Figure 2) without any indication of culture wash out. Then, the amount of YE in the feed was reduced to 5 g L^−1^ (gray curve in Figure 2), and *D* was set to 0.65 h^−1^ to prevent a potential wash out. At process time 10 day, *D* was increased to 0.72 h^−1^ without detectable loss of biomass. After 11 day, a sample of the biomass was harvested and preserved in glycerol stocks, and the process was stopped. The subsequent two continuous processes (c.f., Figure 2) were conducted in the same way: whenever stable process conditions were reached (indicated by off-gas analysis), *D* was increased and the amount of YE was decreased afterward (16 day: 3 g L^−1^, 28 day: 1.5 g L^−1^). Notably, the final subpopulation of the preceding process always served as the inoculum for the subsequent experiment. In the fourth process starting at process time 41 day and with 1.5 g YE L^−1^, a maximum growth rate of 0.76 h^−1^ was set at approx. 42 day which had to be reduced to 0.7 h^−1^ due to the wash out of the culture. Thereupon, YE was reduced several times to 1 g L^−1^ (48 day), 0.5 g L^−1^ (52 day), and 0.25 g L^−1^ (58 day). During this process the dilution rate had to be lowered repeatedly to *D* = 0.67 h^−1^ to avoid a wash-out. After 62 day (1,490 generations), YE could be omitted completely from the feed medium. Hence, for the following 12 day of the last process, the strain was cultured in minimal medium with glucose as sole carbon source. The final evolved strain was able to sustain a stable growth rate of around 0.52 h^−1^. Consequently, five potentially evolved strains were harvested over the course of the combined processes: EVO1 (214 generations), EVO2 (520 generations), EVO3 (984 generations), EVO4 (1,448 generations), and EVO5 (1,700 generations).

### Mutations in Evolved Strain EVO5 and Growth Characterization

For the final evolved strain EVO5, whole-genome sequencing was applied to identify mutations in its entire genome (Table 1) revealing a total of 10 mutations. Among them were mutations in cg0655 (*rpoA*) encoding the DNA-directed RNA polymerase alpha subunit, cg2103 (*dtxR*) encoding the iron-dependent regulator DtxR, cg2831 (*ramA*) that encodes the global carbon regulator RamA, and cg2935 (*nanP*) coding for a neuraminidase. Additionally, mutations appeared in the intergenic region upstream of cg3285 (*copR*, encoding a putative response regulator), in cg2069 (*psp1*, coding for a putative secreted protein) in genes cg2293 and cg2468 that encode a putative indole-3-glycerol phosphate synthase and the permease component of a branched-chain amino acid ABC-type transport system, respectively, as well as in genes cg2067 and cg2504 that both code for hypothetical proteins.

**Table 1:** Mutations of *C. glutamicum* strain EVO5 deduced from the final continuous evolution processes (c.f., Figure 2).

Locus	Gene	Variant	Mutation/Deletion	Annotation
Pos. 3,168,391		Deletion	G	Intergenic region upstream of *copR*
cg2831	*ramA*	Mutation	S101C (G**G**A → G**C**A)	Bacterial regulatory protein, LuxR family
cg0655	*rpoA*	Mutation	S280F (G**G**A → G**A**A)	DNA-directed RNA polymerase alpha subunit
cg2067		Mutation	L91 V (**T**TG → **G**TG)	Hypothetical protein
cg2069	*psp1*	Mutation	P511 (CC**T** → CC**G**)	Putative secreted protein
cg2103	*dtxR*	Mutation	R103H (C**G**C → C**A**C)	Iron-dependent regulatory protein
cg2293		Deletion, Frameshift	T (nt 139), AA55 stop	Putative indole-3-glycerol phosphate synthase
cg2468		Mutation	N97D (**A**AC → **G**AC)	Branched-chain amino acid ABC-type transport system, permease component
cg2504		Mutation	R265 (CG**T** → CG**C**)	Hypothetical protein
cg2935	*nanP*	Mutation	A265 (GC**C** → GC**T**)	Neuraminidase

*The positions of mutations are given in relation to the start codon of respective open reading frames from each gene. EVO5 was harvested after 1,700 generations in solely glucose-supplemented CGXII minimal medium [1% (w/v) glucose]*.

Characterizations of *C. glutamicum* WT, EVO5, and re-engineered mutant strain *C. glutamicum* reRamA (Cg reRamA) were conducted in independent triplicates applying bioreactor batch cultivations. All strains were cultivated under the same conditions in minimal medium CGXII with 2% (w/v) glucose and without PCA. The kinetic parameters determined for each of these strains are listed in Table 2. The WT exhibited a growth rate of 0.34 ± 0.03 h^−1^. EVO5 was characterized by a significantly enhanced growth rate of 0.54 ± 0.01 h^−1^. This is an improvement of 58% toward the WT performance. Cg reRamA also revealed an elevated growth rate of 0.38 ± 0.03 h^−1^ compared to the WT. Glucose consumption (*q*_Glc_) of EVO5 increased by 62% and that of Cg reRamA by 20% compared to the WT (0.64 ± 0.06 g g^−1^ h^−1^). EVO5 showed likewise proportionally increased respiratory rates of *q*O_2_ = 9.14 ± 0.31 mmol g^−1^ h^−1^ and *q*CO_2_ = 9.70 ± 0.18 mmol g^−1^ h^−1^ while exhibiting a WT-like biomass-substrate yield of 0.52 ± 0.01 g g^−1^. While respiratory rates of Cg reRamA were also proportionally increased with the growth rate (*q*O_2_ = 7.04 ± 0.56 mmol g^−1^ h^−1^; *q*CO_2_ = 6.93 ± 0.55 mmol g^−1^ h^−1^), its biomass-substrate yield was below that of the WT (0.49 ± 0.01 g g^−1^).

**Table 2:** Summary of kinetic parameters determined for *C. glutamicum* WT, *C. glutamicum* reRamA (Cg reRamA), and EVO5 grown in CGXII minimal medium supplemented with 2% (w/v) glucose.

Strain	*μ*	*q*_Glc_	*Y*_XS_	*q*O_2_	*q*CO_2_
	h^−1^	g g^−1^ h^−1^	g g^−1^	mmol g^−1^ h^−1^	mmol g^−1^ h^−1^
WT	0.34 ± 0.03	0.64 ± 0.06	0.53 ± 0.01	6.06 ± 0.48	6.17 ± 0.65
Cg reRamA	0.38 ± 0.03	0.77 ± 0.04	0.49 ± 0.01	7.04 ± 0.56	6.93 ± 0.55
EVO5	0.54 ± 0.01	1.03 ± 0.01	0.52 ± 0.01	9.14 ± 0.31	9.70 ± 0.18

*Bioreactor batch cultivations were performed at 30°C, 1.5 bar, and pH 7.4 in biological triplicates. Values represent the statistical mean ± standard deviation. μ, exponential growth rate; q_Glc_, glucose consumption rate; Y_XS_, biomass-substrate yield; qO_2_, oxygen consumption rate; qCO_2_, carbon dioxide emission rate*.

**Figure 3:**
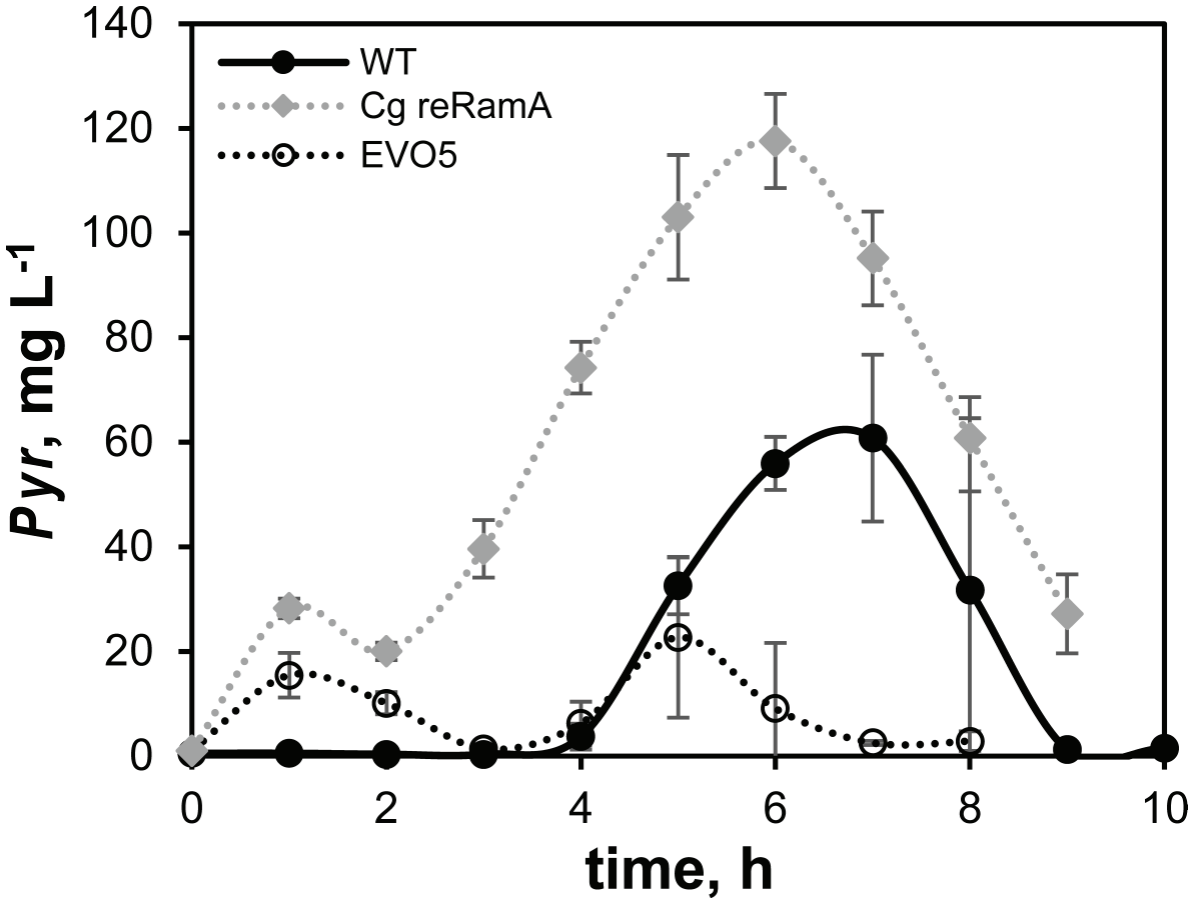
Pyruvate (Pyr) accumulation during exponential growth phases of strains *C. glutamicum* (WT), Cg reRamA, and EVO5 cultivated in CGXII minimal medium supplemented with 2% (w/v) glucose in a bioreactor. Bioreactor batch cultivations were performed at 30°C, 1.5 bar, and pH 7.4 in biological triplicates. Error bars represent ± standard deviation from the mean.

Only pyruvate could be detected as by-product of the batch cultivations. Pyruvate concentration in the WT samples (black filled circles, Figure 3) started to rise between 3 and 4 h and peaked at about 7 h with a concentration of 60 mg L^−1^. Afterward, this amount was completely consumed until the end of the processes (9–10 h). EVO5 (black open circles) built up concentrations of approx. 15 mg L^−1^ pyruvate in the medium during the first 3 h of the cultivation. In contrast to the WT, concentrations of pyruvate did not increase markedly and peaked at 5 h (23 mg L^−1^). The by-product was completely consumed by EVO5 at the end of the cultivation. Cg reRamA accumulated about twice as much pyruvate than the WT (120 mg L^−1^ at process time 6 h) and a residual amount of 30 mg L^−1^ was still detectable after the end of the exponential growth phase (process time 9 h).

## Discussion

### Adaptive Laboratory Evolution Concept

The aim of the ALE experiments was to increase the growth rate of the industrial workhorse *C. glutamicum* by applying a series of increasingly challenging dilution conditions in continuously operated bioreactors. Concomitantly, the fraction of YE feeding was reduced finally adding an additional selection pressure. After about 2.5 months of total process time reflecting 1,700 generations, the evolution mutant EVO5 was obtained. This strain exhibited a growth rate of 0.54 h^−1^ which represents an improvement of 58% compared to the WT strain.

Recently, two independent ALE studies also evolved *C. glutamicum* WT with the purpose to improve the growth rate (Pfeifer et al., 2017; Wang et al., 2018). Both approaches followed a different ALE concept by employing repeated batches passaging stationary cells from glucose-depleted minimal medium to glucose-rich medium. Accordingly, cells repetitively encountered highly varying growth environments that were characterized by increasing biomass concentrations, decreasing glucose and oxygen availability, and changing pH over the respective process times (Seletzky et al., 2007) Intrinsically, such conditions are vulnerable to induce by-product formation either due to overflow metabolism (Paczia et al., 2012) or due to oxygen limitation (Lange et al., 2018). As a consequence, such effects may impose additional selection constraints, e.g., selecting for those cells that may withstand said conditions at best, e.g., by co-consuming by-products the fastest. To avoid such additional selection pressures, this study used strictly controlled bioreactor cultivations (pH 7.4; pO_2_ > 30%) operating in continuous chemostat mode. Notably, the fine-tuned controller enabled dilution rate (*D*) settings with a precision of 0.01 h^−1^ close to the growth maximum of the culture. In contrast to batch ALE selections, the continuous mode regime counteracts by-product accumulation by diluting such substances. Besides, operating at a high dilution rate obviously discriminates for the fastest-growing sub-populations while slow growers are washed out (Gostomski et al., 1994). As a side effect, fast-growing subpopulations do not have to compete for nutrients with slow-growers as it is the case in, e.g., (fed-) batch cultivations. Consequently, the described ALE concept exclusively selects for fast-growing subpopulations. Continuous growth selection experiments somewhat differ from batch-wise approaches. Apparently, cells undergo changing nutrient supply in batch cultures, which is only happening in continuous selections when growth kinetics of the selected population changes. Intrinsically, continuous cultures prevent the occurrence of stationary growth that may occur intermediately in sequential batch-wise growth selections. In the continuous experiments of this study, a high number of generations of *C. glutamicum* was produced over the course of the experiments (680 generations per month) which increases the selection speed of an ALE process.

### Adaptive Laboratory Evolution Medium and Protocatechuate Deficiency

Unlike other approaches, CGXII medium supplemented with YE was initially used as growth medium. Importantly, the amount of YE was gradually reduced while dilution rates increased. As a consequence, a double selection pressure was imposed on *C. glutamicum*: first to compete with the steadily rising dilution rates by increasing growth and second to increase the own biosynthesis of amino acids because the external supply diminished. Using large amounts of YE liberates the cells from their necessity to produce amino acids from glucose. Accordingly, related demands of precursors, NADPH and ATP, are reduced under amino acid supplementation. Graf et al. (2018) have outlined that *C. glutamicum* WT possesses a maximum glucose uptake rate (*q*_Glc,max_) of approx. 0.0275 C-mol g_CDW_^−1^ h^−1^ which equals 0.825 g_Glc_ g_CDW_^−1^ h^−1^ if synthetic CGXII medium is supplemented with up to 10 g brain-heart-infusion per liter. Further supplementation of the complex media decreased *q*_Glc_, while the total carbon consumption *q*_C_ enhanced proportionally with *μ* yielding a constant biomass-carbon yield of 18.47 g C-mol^−1^. This phenotype hinted at a maximum catabolic capacity to metabolize glucose for providing precursors of amino acid formation. The selection strategy of our study tackles this fundamental problem. Starting with high YE fractions while concomitantly challenging growth was purposely installed to direct evolution to targets controlling amino acid use and not the precursor supply for amino acid formation. Accordingly, it was expected that genes coding for basic regulatory functions may be evolved rather than genes encoding reactions of the central metabolism. Further reducing the YE fraction while keeping growth selection pressure high was expected to shift selection pressure to genes of central metabolism and related regulons. As such, the selection scenario fundamentally differs from those of previous studies and gives rise to the identification of different mutation sets, as shown in the next paragraph.

Besides, another fact is worth noticing: the growth medium did not contain any PCA. Liebl et al. (1989) had shown that aromatic compounds such as PCA or catechol boost growth of *C. glutamicum* anticipating the facilitated iron acquisition as the key driver. Own studies confirmed that *C. glutamicum* WT only achieved *μ* = 0.33 ± 0.02 h^−1^ in PCA-deficient media, whereas 0.45 ± 0.01 and 0.47 ± 0.01 h^−1^ were observed using PCA or catechol, respectively (c.f., Supplementary Figure S2). Following the idea to evolve novel *C. glutamicum* strains for industrial application, the study purposely resisted to add PCA to reduce media costs. Accordingly, this constraint may be qualified as an additional threshold compared to the published strains of Pfeifer et al. (2017) and Wang et al. (2018) which used additional iron chelators, i.e., PCA and catechol, respectively. As indicated in Table 2, the growth selection using CGXII minimal medium [1% (w/v) glucose] without PCA (Buchholz et al., 2014) improved the growth rate by 58%. Previous studies of Pfeifer et al. (2017) and Wang et al. (2018) only achieved improvements of 26 and 42%, notably still requiring iron chelators. With *μ*_EVO5_ = 0.54 h^−1^ EVO5 even outperformed the *C. glutamicum* WT growing on PCA containing medium (*μ*_WT_ = 0.45 h^−1^). Furthermore, supplementation of PCA under did not boost the growth of EVO5 further (c.f., Supplementary Figure S3) which is an interesting target for further research. Summarizing, these findings question the anticipated function on PCA only serving as iron chelator. Another explanation was offered by Shen et al. (2012) who reported that both aromatic compounds can be degraded by *C. glutamicum via* the β-ketoadipate pathway to succinyl-CoA finally fueling the citric acid (TCA) cycle. Unthan et al. (2014) proposed that this effect can explain the improved growth performance in highly diluted environments in presence of PCA. However, such a hypothesis would assume boosting growth of EVO5 after PCA supplementation, which was not observed under the tested conditions. Accordingly, an alternate hypothesis may be valid assigning PCA a regulatory function. Similar observations have been made for numerous siderophores in addition to the iron-chelating properties, but are not yet described for PCA (Johnstone and Nolan, 2015).

### Mutations in EVO5

In comparison to the parental WT strain, the genome of EVO5 revealed a total of 10 mutations (c.f., Table 1) of which seven mutations were supposedly not responsible for the increased growth performance of EVO5 observed under the tested conditions. The deletion of guanin at Pos. 3168391 was in the intergenic region between cg3285 (*copR*) and cg3286 which is 28 bp ahead of the start codon of *copR* and does not interfere with the binding site of CopR between bp −59 and −78 (Schelder et al., 2011). CopR is a (putative) response regulator and part of the copper-responsive two-component system CopRS serving as key regulatory system in *C. glutamicum* for copper ion resistance (Schelder et al., 2011). However, the transcriptional start site of *copR* was not identified yet (Schelder et al., 2011, Pfeifer-Sancar et al., 2013).

Another mutation was a base exchange in the *psp1* gene that did not lead to an amino acid exchange and therefore should not change the protein’s activity. Interestingly, this gene is located in the cryptic prophage element CGP3 (Kalinowski et al., 2003) and was also mutated in the studies of Pfeifer et al. (2017) and Wang et al. (2018). The latter also detected that a 180 kbp fragment of the CGP3 region (cg1890–cg2071) was additionally missing and several mutations were observed in the final part of CGP3 (cg2066–cg2069). Notably, we detected a mutation in cg2067 that is leading to an amino acid exchange in the same region. It seems that mutations in this region are a common occurrence after ALE experiments. Additionally, Baumgart et al. (2013) showed that the removal of this region does not have an adverse effect on growth. Taken together, this makes it likely that mutations in this region do not affect growth and that this mutation is not causative for the improved growth performance.

Nucleotide changes of EVO5 in genes cg2504 and *nanP* did not influence the encoded amino acid either. The gene cg2468, encoding the permease component of a branched-chain amino acid ABC-type transporter, was affected by an amino acid exchange. Possibly, using decreasing amounts of YE as growth supplement in the first ALEs might have provoked this mutation, but it was not lost in the following YE-free phase where minimal medium with glucose as sole carbon source was applied. A deletion of base 139 was observed in cg2293 leading to a frameshift and consequent stop codon at amino acid 55. Since the stop codon occurred at the very beginning of the normally 261 amino acid-long protein, the resulting enzyme (putative indole-3-glycerol phosphate synthase^1^) is most likely not functional.

Three other mutations occurred in genes involved in regulatory processes within EVO5, which is in accordance with the experimental design. They are anticipated to determine the growth phenotype extraordinarily. The RamA protein of *C. glutamicum* was first identified by Cramer and Eikmanns (2007) as a master regulator of acetate metabolism. In the presence of acetate, RamA activates the *pta*-*ack* genes encoding phosphotransacetylase and acetate kinase as well as *aceA*, and *aceB* encoding the enzymes of the glyoxylate shunt isocitrate lyase and malate synthase, respectively. Further studies showed that RamA is a global regulator in the carbon metabolism of *C. glutamicum* and influences genes of carbon uptake, glucose-, ethanol-, and propionate-metabolism, as well as cell wall synthesis (functions of RamA were recently reviewed by Shah et al., 2018). Comparable to our study, the repetitive batch ALE of Wang et al. (2018) triggered a point mutation in the *ramA* gene (yielding RamA^A52V^). The mutation was re-engineered into the WT and into a prophage-free strain (this resulting strain was called LUXR) which led to increased growth rates by 20 and 22%, respectively, compared to the respective parental strains. To test if the mutated RamA^S101C^ obtained in our ALE led to a similar phenotype, we re-engineered the mutation in the WT (called Cg reRamA) and cultivated it under standard batch conditions (CGXII without PCA, 2% (w/v) glucose) in a bioreactor. In contrast to Wang et al. (2018), Cg reRamA exhibited only a slight growth enhancement of 8% compared to the WT-control (*μ* = 0.34 ± 0.03 h^−1^). However, we observed a non-proportional increase of the glucose consumption rate by 20% (WT: *q*_Glc_ = 0.64 ± 0.06 g g^−1^ h^−1^) that was comparable to rates determined by Wang et al. (2018). Correspondingly, a two-tailed *t*-test showed that the biomass-substrate yield of Cg reRamA was significantly smaller compared to the WT (Cg reRamA: 0.49 ± 0.01 g_CDW_ g_Glc_^−1^; WT: 0.53 ± 0.01 g_CDW_ g_Glc_^−1^, c.f., Table 2). The main reason was found by studying the by-product formation of pyruvate. Indeed, Cg reRamA and the WT secreted rising amounts of pyruvate until the mid-exponential growth phase (c.f., Figure 3, WT-maximum: 60 mg L^−1^, Cg reRamA-maximum: 120 mg L^−1^).

However, Cg reRamA secreted twice as much of the organic acid and was not able to re-consume pyruvate completely. Since pyruvate marks the intersection between glycolysis and TCA, this metabolic overflow phenotype indicated unbalanced fluxes between both central catabolic routes. Apparently, the enhanced glucose consumption rates of Cg reRamA mirrored alleviated glycolytic fluxes, which could not be fueled into the TCA but yielded increased pyruvate secretion instead. This finding is in accordance with results of Wang et al. (2018) who outlined that the *ramA* mutation in LUXR led to an up-regulation of glycolytic genes concomitantly with a slight decrease of TCA activity. The *ramA* point mutations identified by Wang et al. (2018) and in our study are both not located in the DNA-binding site of the protein (HTH motif between positions 214–274 at the C terminus), but in the GAF-2 domain (amino acid positions 8 and 146) at the N terminus (Cramer and Eikmanns, 2007). As summarized in Shah et al. (2018), this domain is (among other functions) associated with gene regulation in bacteria. Cramer and Eikmanns (2007) showed that *C. glutamicum* without HTH-motif in RamA was not able to grow on acetate as sole carbon source. However, the observed GAF-2-mutation in Cg reRamA also impaired growth in CGXII minimal medium with acetate as sole carbon source (c.f., Supplementary Figure S1), but did not impair this growth entirely. Besides, the specific mutation S101C in RamA in EVO5 provoked a non-proportional *q*_Glc_ increase, which was not observed in the RamA-mutants of Wang et al. (2018). A mutation occurred in the gene encoding DtxR, the central regulator of the iron metabolism in *C. glutamicum* (Wennerhold and Bott, 2006). Interestingly, both, DtxR and the previously discussed RamA, have an influence on the regulation of the succinate dehydrogenase operon *sdhCAB* under standard growth conditions (Brune et al., 2006; Bussmann et al., 2009; Auchter et al., 2011) Besides, the *dtxR* mutation may also mirror the cellular need to improve iron availability during growth as outlined by Rolfe et al. (2012).

Another interesting mutation occurred in the gene *rpoA* encoding the alpha-subunit of the RNA polymerase (RNAP). The alpha subunit is part of the RNAP core enzyme and has been described to interact with regulators and to be able to alter transcription initiation (Ross et al., 1993; Dangi et al., 2004; Kim et al., 2012). The alpha-subunit has two domains, the alpha N-terminal domain, essential for RNAP assembly and basal transcription (αNTD), and the alpha C-terminal domain (αCTD), which is interacting with transcriptional regulators and promoter DNA to regulate transcription (Busby and Ebright, 1999; Browning and Busby, 2004). It has been reported that mutations in the αNTD region caused temperature sensitivity (Ishihama et al., 1980), while several mutations in the αCTD induced metabolic changes (Jafri et al., 1995; Klein-Marcuschamer et al., 2009). The present mutation altered the encoded amino acid at position 280, which places it near the center of the αCTD. Because of the range of phenotype changes elicited by mutations in this region, the alpha-subunit of the RNAP was previously proposed as an interesting target for metabolic engineering when global transcriptional changes are required (Klein-Marcuschamer et al., 2009). It is therefore likely that the present mutation in RpoA caused a wide-reaching change in gene expression and might be responsible for the further growth rate improvements. A comparable observation was made by LaCroix et al. (2015) in an *E. coli* ALE study. Among other mutations, reproducible mutations in the gene *rpoB* were observed that together with *rpoC* encodes the beta chain of the RNA polymerase. Even though the mutations manifested at different positions in *rpoB*, each apparently improved *E.coli*’s growth rate.

Summarizing, a set of 10 distinct mutations has been identified in EVO5 of which six supposedly had no influence on the growth performance. Strikingly, three of the residual four mutated genes that most likely were responsible for the growth improvement are global regulators. This accredits the motivation of performing the selection study with the dual pressure to reduce complex media and iron chelator components while challenging growth rates. As such, findings differ from the mutations identified by Pfeifer et al. (2017) in central metabolism genes such as *pyk* (encoding pyruvate kinase; Ozaki and Shiio, 1969; Jetten et al., 1994) and *fruK* (or *pfkB*; cg2119), coding for 1-phosphofructokinase; Sugimoto and Shiio, 1989). Apparently, *C. glutamicum* found ways to adapt to the challenging growth conditions by changing the global regulatory responses, which even allowed the strain to grow without extra iron chelator addition.

### Preliminary Characterization of EVO5

Preliminary evaluations of the EVO5 mutant strain with respect to PCA sensitivity as well as acetate, lactate, and gluconate consumption are indicated in the appendix (c.f., Supplementary Table S2). Shaking flask experiments were performed and in accordance with the bioreactor cultivations (c.f., Section “Adaptive Laboratory Evolution Medium and PCA Deficiency”; inoculation density: OD_600_ 1), EVO5 shows a maximum growth rate of 0.63 h^−1^ without PCA, superior to the WT growth of 0.36 h^−1^. Under the same conditions, addition of PCA to the medium does not further accelerate growth of EVO5 while it boosts the growth of *C. glutamicum* WT to 0.55 h^−1^, which is still lower than the one exhibited by EVO5. Employing an inoculation density of OD 0.5 and supplementing PCA slightly enhanced *μ* of EVO5 from 0.62 h^−1^ (without PCA) to 0.66 h^−1^. This growth stimulating effect of PCA in combination with low inoculation densities was previously described for the WT (Unthan et al., 2014) and will be the topic of future research for EVO5. With regard to alternative carbon sources as growth substrate, *C. glutamicum* WT and EVO5 exhibit significantly reduced growth rates on acetate as sole carbon source. In particular, growth of EVO5 is lowered to 0.23 h^−1^, which is less than WT performance. Apparently, the mutations of EVO5 interact with the strains capacity to metabolize acetate. *C. glutamicum* displays severely reduced growth on lactate as sole carbon source similar to the case of acetate as sole carbon source. While growth rate of the WT is faster than that of EVO5 under these conditions, both rates are the lowest in the experimental study. Although gluconate metabolism differs from glucose uptake and metabolism, its substrate conversion is more comparable to that of glucose than to that of acetate and lactate as alternate carbon sources. Consequently, it may not surprise that gluconate-based growth of EVO5 is faster than of the WT, by trend. Biomass yields of both strains are fairly similar.

## Conclusion

The installation of a double selection pressure comprising reduction of complex medium supplements and steady increase of growth yielded the successful selection of *C. glutamicum* EVO 5. With *μ* = 0.54 h^−1^, the strain outnumbered the WT performance by 58%, noteworthy without using well-known boosters such as PCA. Consequently, the strain is easy-to-transfer in industrial conditions, which allows its use as a novel production platform for growth-coupled products. So far, the relatively low growth rate of *C. glutamicum* restricted its application for partially growth-coupled products only requiring moderate growth associated production kinetics. Now, other applications are in reach further exploiting the native traits of the *C. glutamicum* chassis. Additionally, 10 mutations of which three are regulator genes identified in the growth selections provide a fruitful basis for systems metabolic engineering boosting strain kinetics even further.

## Data Availability

All datasets generated for this study are included in the manuscript and/or the Supplementary Files.

## Author Contributions

MG and TH designed the study, analyzed the datasets, and drafted the manuscript. MG, TH, and JH-B carried out the bioreactor experiments, and AF designed the control scheme for the continuous process mode. FM constructed strain *C. glutamicum* reRamA. AB performed characterization studies of *C. glutamicum* WT and EVO5 in shaking flasks. MP performed sequencing of the *C. glutamicum* WT and evolution strain EVO5. JH-B, AF, FM, AB, AN, MP, JK, and BB analyzed the datasets and corrected the manuscript. RT conceived the study and corrected the manuscript. All authors read and approved the final manuscript.

### Conflict of Interest Statement

The authors declare that the research was conducted in the absence of any commercial or financial relationships that could be construed as a potential conflict of interest.

## Footnotes

^1^Information retrieved on 2 December 2018 from CoryneRegNet: www.coryneregnet.de

## References

[ref1] Auchter M. Cramer A. Hüser A. Rückert C. Emer D. Schwarz P.. (2011). RamA and RamB are global transcriptional regulators in *Corynebacterium glutamicum* and control genes for enzymes of the central metabolism. J. Biotechnol. 154, 126–139. 10.1016/j.jbiotec.2010.07.001, PMID: 20620178

[ref2] Bäumchen C. Knoll A. Husemann B. Seletzky J. Maier B. Dietrich C.. (2007). Effect of elevated dissolved carbon dioxide concentrations on growth of *Corynebacterium glutamicum* on d-glucose and l-lactate. J. Biotechnol. 128, 868–874. 10.1016/j.jbiotec.2007.01.001, PMID: 17275119

[ref3] Baumgart M. Unthan S. Rückert C. Sivalingam J. Grünberger A. Kalinowski J.. (2013). Construction of a prophage-free variant of *Corynebacterium glutamicum* ATCC 13032 for use as a platform strain for basic research and industrial biotechnology. Appl. Environ. Microbiol. 79, 6006–6015. 10.1128/AEM.01634-13, PMID: 23892752 PMC3811366

[ref4] Becker J. Gießelmann G. Hoffmann S. L. Wittmann C. (2016). “*Corynebacterium glutamicum* for sustainable bioproduction: from metabolic physiology to systems metabolic engineering” in Synthetic biology – Metabolic engineering. Advances in biochemical engineering/biotechnology. Vol. 162, eds. Zhao H. Zeng A. P. (Cham: Springer), 217–263. 10.1007/10_2016_21 27872959

[ref5] Becker J. Wittmann C. (2012). Bio-based production of chemicals, materials and fuels – *Corynebacterium glutamicum* as versatile cell factory. Curr. Opin. Biotechnol. 23, 631–640. 10.1016/j.copbio.2011.11.012, PMID: 22138494

[ref6] Becker J. Wittmann C. (2018). From systems biology to metabolically engineered cells – an omics perspective on the development of industrial microbes. Curr. Opin. Microbiol. 45, 180–188. 10.1016/j.mib.2018.06.001, PMID: 30172106

[ref7] Blombach B. Buchholz J. Busche T. Kalinowski J. Takors R. (2013). Impact of different CO_2_/HCO^3−^ levels on metabolism and regulation in *Corynebacterium glutamicum*. J. Biotechnol. 168, 331–340. 10.1016/j.jbiotec.2013.10.005, PMID: 24140290

[ref8] Browning D. F. Busby S. J. W. (2004). The regulation of bacterial transcription initiation. Nat. Rev. Microbiol. 2, 57–65. 10.1038/nrmicro787 15035009

[ref9] Brune I. Werner H. Hüser A. T. Kalinowski J. Pühler A. Tauch A. (2006). The DtxR protein acting as dual transcriptional regulator directs a global regulatory network involved in iron metabolism of *Corynebacterium glutamicum*. BMC Genomics 7, 1–19. 10.1186/1471-2164-7-21 16469103 PMC1382209

[ref10] Buchholz J. Graf M. Blombach B. Takors R. (2014). Improving the carbon balance of fermentations by total carbon analyses. Biochem. Eng. J. 90, 162–169. 10.1016/j.bej.2014.06.007

[ref11] Busby S. Ebright R. H. (1999). Transcription activation by catabolite activator protein (CAP). Behav. Brain Res. 99, 153–167. 10.1016/S0166-4328(98)00101-6, PMID: 10550204

[ref12] Bussmann M. Emer D. Hasenbein S. Degraf S. Eikmanns B. J. Bott M. (2009). Transcriptional control of the succinate dehydrogenase operon *sdhCAB* of *Corynebacterium glutamicum* by the cAMP-dependent regulator GlxR and the LuxR-type regulator RamA. J. Biotechnol. 143, 173–182. 10.1016/j.jbiotec.2009.06.025, PMID: 19583988

[ref13] Cramer A. Eikmanns B. J. (2007). RamA, the transcriptional regulator of acetate metabolism in *Corynebacterium glutamicum*, is subject to negative autoregulation. J. Mol. Microbiol. Biotechnol. 12, 51–59. 10.1128/JB.01061-06 17183211

[ref14] Dangi B. Gronenborn A. M. Rosner J. L. Martin R. G. (2004). Versatility of the carboxy-terminal domain of the α subunit of RNA polymerase in transcriptional activation: use of the DNA contact site as a protein contact site for MarA. Mol. Microbiol. 54, 45–59. 10.1111/j.1365-2958.2004.04250.x, PMID: 15458404

[ref15] Dettman J. R. Rodrigue N. Melnyk A. H. Wong A. Bailey S. F. Kassen R. (2012). Evolutionary insight from whole-genome sequencing of experimentally evolved microbes. Mol. Ecol. 21, 2058–2077. 10.1111/j.1365-294X.2012.05484.x, PMID: 22332770

[ref16] Dragosits M. Mattanovich D. (2013). Adaptive laboratory evolution – principles and applications for biotechnology. Microb. Cell Factories 12:64. 10.1186/1475-2859-12-64, PMID: 23815749 PMC3716822

[ref17] Feist A. M. Zielinski D. C. Orth J. D. Schellenberger J. Herrgard M. J. Palsson B. O. (2009). Model-driven evaluation of the production potential for growth-coupled products of *Escherichia coli*. Metab. Eng. 12, 173–186. 10.1016/j.ymben.2009.10.003 19840862 PMC3125152

[ref18] Follmann M. Ochrombel I. Krämer R. Trötschel C. Poetsch A. Rückert C.. (2009). Functional genomics of pH homeostasis in *Corynebacterium glutamicum* revealed novel links between pH response, oxidative stress, iron homeostasis and methionine synthesis. BMC Genomics 10:621. 10.1186/1471-2164-10-621, PMID: 20025733 PMC2807442

[ref19] Freudl R. (2017). Beyond amino acids: use of the *Corynebacterium glutamicum* cell factory for the secretion of heterologous proteins. J. Biotechnol. 258, 101–109. 10.1016/j.jbiotec.2017.02.023, PMID: 28238807

[ref20] Gibson D. G. (2011). Enzymatic assembly of overlapping DNA fragments. Methods Enzymol. 498, 349–361. 10.1016/B978-0-12-385120-8.00015-2, 21601685 PMC7149801

[ref21] Gordon D. (2003). Viewing and editing assembled sequences using Consed. Curr. Protoc. Bioinformatics 2, 11.2.1–11.2.43. 10.1002/0471250953.bi1102s02 18428695

[ref22] Gostomski P. Mühlemann M. Lin Y. H. Mormino R. Bungay H. (1994). Auxostats for continuous culture research. J. Biotechnol. 37, 167–177. 10.1016/0168-1656(94)90008-6

[ref23] Graf M. Zieringer J. Haas T. Nieß A. Blombach B. Takors R. (2018). Physiological response of *Corynebacterium glutamicum* to increasingly nutrient-rich growth conditions. Front. Microbiol. 9, 1–15. 10.3389/fmicb.2018.02058, 30210489 PMC6123352

[ref24] Grünberger A. van Ooyen J. Paczia N. Rohe P. Schiendzielorz G. Eggeling L.. (2013). Beyond growth rate 0.6: *Corynebacterium glutamicum* cultivated in highly diluted environments. Biotechnol. Bioeng. 110, 220–228. 10.1002/bit.24616, PMID: 22890752

[ref25] Ishihama A. Shimamoto N. Aiba H. Kawakami K. Nashimoto H. Tsugawa A.. (1980). Temperature-sensitive mutations in the α subunit gene of *Escherichia coli* RNA polymerase. J. Mol. Biol. 137, 137–150. 10.1016/0022-2836(80)90321-6, PMID: 6154799

[ref26] Jafri S. Urbanowski M. L. Stauffer G. V. (1995). A mutation in the *rpoA* gene encoding the α subunit of RNA polymerase that affects *metE*-*metR* transcription in *Escherichia coli*. J. Bacteriol. 177, 524–529. 10.1128/jb.177.3.524-529.1995, PMID: 7836282 PMC176623

[ref27] Jetten M. S. Gubler M. E. Lee S. H. Sinskey A. J. (1994). Structural and functional analysis of pyruvate kinase from *Corynebacterium glutamicum*. Appl. Environ. Microbiol. 60, 2501–2507. PMID: 8074528 10.1128/aem.60.7.2501-2507.1994 PMC201676

[ref28] Johnstone T. C. Nolan E. M. (2015). Beyond iron: non-classical biological functions of bacterial siderophores. Dalton Trans. 44, 6320–6339. 10.1039/C4DT03559C, PMID: 25764171 PMC4375017

[ref29] Kalinowski J. Bathe B. Bartels D. Bischoff N. Bott M. Burkovski A.. (2003). The complete *Corynebacterium glutamicum* ATCC 13032 genome sequence and its impact on the production of L-aspartate-derived amino acids and vitamins. J. Biotechnol. 104, 5–25. 10.1016/S0168-1656(03)00154-8, PMID: 12948626

[ref30] Keilhauer C. Eggeling L. Sahm H. (1993). Isoleucine synthesis in *Corynebacterium glutamicum*: molecular analysis of the ilvB-ilvN-ilvC operon. J. Bacteriol. 175, 5595–5603. 10.1128/jb.175.17.5595-5603.1993, PMID: 8366043 PMC206616

[ref31] Kim J. H. Ham S. H. Lee B. R. (2012). Characterization of the RNA polymerase α subunit operon from *Corynebacterium ammoniagenes*. World J. Microbiol. Biotechnol. 28, 669–676. 10.1007/s11274-011-0861-9, PMID: 22806862

[ref32] Kind S. Jeong W. K. Schröder H. Zelder O. Wittmann C. (2010). Identification and elimination of the competing N-acetyldiaminopentane pathway for improved production of diaminopentane by *Corynebacterium glutamicum*. Appl. Environ. Microbiol. 76, 5175–5180. 10.1128/AEM.00834-10, PMID: 20562290 PMC2916474

[ref33] Klein-Marcuschamer D. Santos C. N. S. Yu H. Stephanopoulos G. (2009). Mutagenesis of the bacterial RNA polymerase alpha subunit for improvement of complex phenotypes. Appl. Environ. Microbiol. 75, 2705–2711. 10.1128/AEM.01888-08, PMID: 19251886 PMC2681691

[ref34] LaCroix R. A. Sandberg T. E. O'Brien E. J. Utrilla J. Ebrahim A. Guzman G. I. (2015). Use of adaptive laboratory evolution to discover key mutations enabling rapid growth of *Escherichia coli* K-12 MG1655 on glucose minimal medium. Appl. Environ. Microbiol. 81, 17–30. 10.1128/AEM.02246-14 25304508 PMC4272732

[ref35] Lange J. Münch E. Müller J. Busche T. Kalinowski J. Takors R.. (2018). Deciphering the adaptation of *Corynebacterium glutamicum* in transition from aerobiosis *via* microaerobiosis to anaerobiosis. Genes 9:297. 10.3390/genes9060297, PMID: 29899275 PMC6027265

[ref36] Lee D. H. Palsson B. O. (2010). Adaptive evolution of *Escherichia coli* K-12 MG1655 during growth on a nonnative carbon source, L-1, 2-propanediol. Appl. Environ. Microbiol. 76, 4158–4168. 10.1128/AEM.00373-10, PMID: 20435762 PMC2897412

[ref37] Liebl W. Klamer R. Schleifer K. H. (1989). Requirement of chelating compounds for the growth of *Corynebacterium glutamicum* in synthetic media. Appl. Microbiol. Biotechnol. 32, 205–210. 10.1007/BF00165889

[ref38] Löffler M. Simen J. D. Jäger G. Schäferhoff K. Freund A. Takors R. (2016). Engineering *E. coli* for large-scale production – strategies considering ATP expenses and transcriptional responses. Metab. Eng. 38, 73–85. 10.1016/j.ymben.2016.06.008, PMID: 27378496

[ref39] McCloskey D. Xu S. Sandberg T. E. Brunk E. Hefner Y. Szubin R.. (2018). Adaptation to the coupling of glycolysis to toxic methylglyoxal production in tpiA deletion strains of *Escherichia coli* requires synchronized and counterintuitive genetic changes. Metab. Eng. 48, 82–93. 10.1016/j.ymben.2018.05.012, PMID: 29842925

[ref40] Michalowski A. Siemann-Herzberg M. Takors R. (2017). *Escherichia coli* HGT: engineered for high glucose throughput even under slowly growing or resting conditions. Metab. Eng. 40, 93–103. 10.1016/j.ymben.2017.01.005, PMID: 28110078

[ref41] Mohamed E. T. Wang S. Lennen R. M. Herrgård M. J. Simmons B. A. Singer S. W. (2017). Generation of a platform strain for ionic liquid tolerance using adaptive laboratory evolution. Microb. Cell Factories 16:204. 10.1186/s12934-017-0819-1 PMC5691611 29145855

[ref42] Nielsen J. Keasling J. D. (2016). Engineering cellular metabolism. Cell 164, 1185–1197. 10.1016/j.cell.2016.02.004, PMID: 26967285

[ref43] Nishimura T. Vertès A. A. Shinoda Y. Inui M. Yukawa H. (2007). Anaerobic growth of *Corynebacterium glutamicum* using nitrate as a terminal electron acceptor. Appl. Microbiol. Biotechnol. 75, 889–897. 10.1007/s00253-007-0879-y, PMID: 17347820

[ref44] Ozaki H. Shiio I. (1969). Regulation of the TCA and glyoxylate cycles in *Brevibacterium flavum*: II. Regulation of phosphoenolpyruvate carboxylase and pyruvate kinase. J. Biochem. 66, 297–311. 10.1093/oxfordjournals.jbchem.a129148, PMID: 5348585

[ref45] Paczia N. Nilgen A. Lehmann T. Gätgens J. Wiechert W. Noack S. (2012). Extensive exometabolome analysis reveals extended overflow metabolism in various microorganisms. Microb. Cell Factories 11:122. 10.1186/1475-2859-11-122, PMID: 22963408 PMC3526501

[ref46] Pfeifer E. Gätgens C. Polen T. Frunzke J. (2017). Adaptive laboratory evolution of *Corynebacterium glutamicum* towards higher growth rates on glucose minimal medium. Sci. Rep. 7, 1–14. 10.1038/s41598-017-17014-9 29196644 PMC5711897

[ref47] Pfeifer-Sancar K. Mentz A. Rückert C. Kalinowski J. (2013). Comprehensive analysis of the *Corynebacterium glutamicum* transcriptome using an improved RNAseq technique. BMC Genomics 14:888. 10.1186/1471-2164-14-888, PMID: 24341750 PMC3890552

[ref48] Rolfe M. D. Rice C. J. Lucchini S. Pin C. Thompson A. Cameron A. D. S.. (2012). Lag phase is a distinct growth phase that prepares bacteria for exponential growth and involves transient metal accumulation. J. Bacteriol. 194, 686–701. 10.1128/JB.06112-11, PMID: 22139505 PMC3264077

[ref49] Ross W. Gosink K. K. Salomon J. Igarashi K. Zou C. Ishihama A.. (1993). A third recognition element in bacterial promoters: DNA binding by the alpha subunit of RNA polymerase. Science 262, 1407–1413. 10.1126/science.8248780, PMID: 8248780

[ref50] Rugbjerg P. Feist A. M. Sommer M. O. A. (2018). Enhanced metabolite productivity of *Escherichia coli* adapted to glucose M9 minimal medium. Front. Bioeng. Biotechnol. 6:166. 10.3389/fbioe.2018.00166, PMID: 30483499 PMC6240765

[ref51] Ryall B. Eydallin G. Ferenci T. (2012). Culture history and population heterogeneity as determinants of bacterial adaptation: the adaptomics of a single environmental transition. Microbiol. Mol. Biol. Rev. 76, 597–625. 10.1128/MMBR.05028-11, PMID: 22933562 PMC3429624

[ref52] Sambrook J. (2001). Molecular cloning: A laboratory manual. Cold Spring Harbor, NY: Cold Spring Harbor Laboratory Press.

[ref53] Sandberg T. E. Long C. P. Gonzalez J. E. Feist A. M. Antoniewicz M. R. Palsson B. O. (2016). Evolution of *E. coli* on [U-13C] glucose reveals a negligible isotopic influence on metabolism and physiology. PLoS One 11:e0151130. 10.1371/journal.pone.0151130, PMID: 26964043 PMC4786092

[ref54] Schäfer A. Tauch A. Jäger W. Kalinowski J. Thierbach G. Pühler A. (1994). Small mobilizable multi-purpose cloning vectors derived from the *Escherichia coli* plasmids pK18 and pK19: selection of defined deletions in the chromosome of *Corynebacterium glutamicum*. Gene 145, 69–73. 10.1016/0378-1119(94)90324-7, PMID: 8045426

[ref55] Schelder S. Zaade D. Litsanov B. Bott M. Brocker M. (2011). The two-component signal transduction system CopRS of *Corynebacterium glutamicum* is required for adaptation to copper-excess stress. PLoS One 6:e22143. 10.1371/journal.pone.0022143 21799779 PMC3140484

[ref56] Seletzky J. M. Noak U. Fricke J. Welk E. Eberhard W. Knocke C.. (2007). Scale-up from shake flasks to fermenters in batch and continuous mode with *Corynebacterium glutamicum* on lactic acid based on oxygen transfer and pH. Biotechnol. Bioeng. 98, 800–811. 10.1002/bit.21359, PMID: 17318907

[ref57] Shah A. Blombach B. Gauttam R. Eikmanns B. J. (2018). The RamA regulon: complex regulatory interactions in relation to central metabolism in *Corynebacterium glutamicum*. Appl. Microbiol. Biotechnol., 102:5901. 10.1007/s00253-018-9085-3 29804137

[ref58] Shen X. H. Zhou N. Y. Liu S. J. (2012). Degradation and assimilation of aromatic compounds by *Corynebacterium glutamicum*: another potential for applications for this bacterium? Appl. Microbiol. Biotechnol. 95, 77–89. 10.1007/s00253-012-4139-4, PMID: 22588501

[ref59] Stella R. G. Wiechert J. Noack S. Frunzke J. (2019). Evolutionary engineering of *Corynebacterium glutamicum*. Biotechnol. J. 1800444. 10.1002/biot.201800444, PMID: 30927493

[ref60] Straathof A. J. (2013). Transformation of biomass into commodity chemicals using enzymes or cells. Chem. Rev. 114, 1871–1908. 10.1021/cr400309c: 23987659

[ref61] Sugimoto S. I. Shiio I. (1989). Fructose metabolism and regulation of 1-phosphofructokinase and 6-phosphofructokinase in *Brevibacterium flavum*. Agric. Biol. Chem. 53, 1261–1268. 10.1080/00021369.1989.10869488

[ref300] Tauch A. Kirchner O. Löffler B. Götker S. Pühler A. Kalinowski J. (2002). Efficient Electrotransformation of Corynebacterium diphtheriae with a Mini-Replicon Derived from the Corynebacterium glutamicum Plasmid pGA1. Curr. Microbiol. 45, 362–367. 10.1007/s00284-002-3728-3, PMID: 12232668

[ref62] Tremblay P. L. Höglund D. Koza A. Bonde I. Zhang T. (2015). Adaptation of the autotrophic acetogen *Sporomusa ovata* to methanol accelerates the conversion of CO_2_ to organic products. Sci. Rep. 5:16168. 10.1038/srep16168, PMID: 26530351 PMC4632017

[ref63] Unthan S. Grünberger A. van Ooyen J. Gätgens J. Heinrich J. Paczia N.. (2014). Beyond growth rate 0.6: what drives *Corynebacterium glutamicum* to higher growth rates in defined medium. Biotechnol. Bioeng. 111, 359–371. 10.1002/bit.25103, PMID: 23996851

[ref64] van der Rest M. Lange C. Molenaar D. (1999). A heat shock following electroporation induces highly effcient transformation of *Corynebacterium glutamicum* with xenogeneic plasmid DNA. Appl. Microbiol. Biotechnol. 52, 541–545. 10.1007/s002530051557, PMID: 10570802

[ref65] Wang Z. Liu J. Chen L. Solem C. Jensen P. R. (2018). Alterations in the transcription factors GntR1 and RamA enhance the growth and central metabolism of *Corynebacterium glutamicum*. Metab. Eng. 48, 1–12. 10.1016/j.ymben.2018.05.004, PMID: 29753071

[ref66] Wendisch V. F. Jorge J. M. Pérez-García F. Sgobba E. (2016). Updates on industrial production of amino acids using *Corynebacterium glutamicum*. World J. Microbiol. Biotechnol. 32:105. 10.1007/s11274-016-2060-1 27116971

[ref67] Wendisch V. F. Mindt M. Pérez-García F. (2018). Biotechnological production of mono-and diamines using bacteria: recent progress, applications, and perspectives. Appl. Microbiol. Biotechnol. 102, 3583–3594. 10.1007/s00253-018-8890-z 29520601

[ref68] Wennerhold J. Bott M. (2006). The DtxR regulon of *Corynebacterium glutamicum*. J. Bacteriol. 188, 2907–2918. 10.1128/JB.188.8.2907-2918.2006, PMID: 16585752 PMC1446976

[ref69] Wieschalka S. Blombach B. Bott M. Eikmanns B. J. (2013). Bio-based production of organic acids with *Corynebacterium glutamicum*. Microb. Biotechnol. 6, 87–102. 10.1111/1751-7915.12013, PMID: 23199277 PMC3917452

