# Supplementary material for: Continuous Adaptive Evolution of a Fast-Growing Corynebacterium glutamicum Strain Independent of Protocatechuate
Source: Front Microbiol. 2019 Aug 6;10:1648. doi: 10.3389/fmicb.2019.01648 (PMC6691914; doi:10.3389/fmicb.2019.01648)
Supplement: Supplementary file 1 [file Table_1.DOCX]

Supplementary Material

Continuous adaptive evolution of a fast-growing *Corynebacterium glutamicum* strain independent of protocatechuate

Michaela Graf^1^, Thorsten Haas^1^, Felix Müller^1^, Anina Buchmann^1^, Julia Harm-Bekbenbetova^1^, Andreas Freund^1^, Alexander Nieß^1^, Marcus Persicke^2^, Jörn Kalinowski^2^, Bastian Blombach^1,3^, Ralf Takors^1*^

^1^Institute of Biochemical Engineering, University of Stuttgart, Stuttgart, Germany

^2^Center for Biotechnology (CeBiTec), Bielefeld University, Bielefeld, Germany

^3^Microbial Biotechnology, Campus Straubing for Biotechnology and Sustainability, Technical University of Munich, Straubing, Germany

*** Correspondence:**Prof. Dr.-Ing. Ralf Takors
takors@ibvt.uni-stuttgart.de

# Figures

**Supplementary figure 1.** Growth of *C. glutamicum* ATCC 13032 wild type (WT) and derived strain Cg ReRamA with point mutation in *ramA* (*cg2831*) S101C in CGXII minimal medium (w/o PCA, Buchholz et al., 2014) with 1 % (w/v) acetate as sole carbon source in shaking flasks (30 °C, 120 rpm), n = 3.

**Supplementary figure 2.** Growth of *C. glutamicum* ATCC 13032 wild type (WT) in CGXII minimal medium (Buchholz et al., 2014) with 2 % (w/v) glucose as sole carbon source and optional supplementation 0.1 mM protocatechuate (PCA) or catechol. Cultivations were performed in bioreactors (30 °C, pH = 7.4, pO_2_ > 30 %), n = 3.

**Supplementary figure 3.** Growth of *C. glutamicum* ATCC 13032 EVO5 in CGXII minimal medium (Buchholz et al., 2014) with 2 % (w/v) glucose as sole carbon source and optional supplementation of 30 mg L^-1^ protocatechuate (PCA). Cultivations were performed in bioreactors (30 °C, pH = 7.4, pO_2_ > 30 %), n = 3 (w/o PCA), n = 1 (w/ PCA).

# Tables

Supplementary table 1. Oligonucleotides used in this study.

| **Primer** | **Sequence (5’**→ **3’)** |
| --- | --- |
| **Amplification** |  |
| Fw_ramA-flank1 | *aacagctatgaccatgattacgcc*aagcttGGGGGTTAACTACCTCTTCGG |
| Rev_ramA-flank1 | *cgacaat*G**C**AATGAAGGCCC |
| Fw_ramA-flank2 | *GGGCCTTCATT***G***C*attgtcg |
| Rev_ramA-flank2 | *agtgaattcgagctcggtacccggggatccTTAAGGCAGTGCGCCGATC* |
| **Sequencing** |  |
| Fw_pK19-seq | CAGGCTTTACACTTTATGC |
| Rev_pK19-seq | CCCATCTCTTCAGCAGC |
| Fw_ramA-seq | *GTTGCAGGACAATCGCC* |
| Rev_ramA-seq | *CCCATCTCTTCAGCAGC* |

Supplementary table 2. Summary of growth parameters determined for *C. glutamicum* wild type (WT) and the mutant strain EVO5 grown in CGXII minimal medium containing 2 % (w/v) of the indicated carbon source, respectively. All cultivations were performed in shaking flasks at 30°C and 120 rpm. Values represent the statistical mean $\boldsymbol{\pm}$ standard deviation from biological triplicates.

| **Carbon source** | **Strain** | **Initial OD_600_ (0 h)** | **Final OD_600_ (24 h)** | **Exponential growth rate *µ*_,_ h^-1^** |
| --- | --- | --- | --- | --- |
| Glucose | WT | 0.48 ± 0.01 | 35.33 ± 1.89 | 0.37 ± 0.02 |
|  | EVO5 | 0.48 ± 0.00 | 36.00 ± 1.41 | 0.62 ± 0.00 |
| Glucose + 30 mg L^-1^ PCA | WT | 0.51 ± 0.01 | 35.33 ± 0.82 | 0.50 ± 0.01 |
|  | EVO5 | 0.49 ± 0.00 | 37.33 ± 0.47 | 0.66 ± 0.01 |
| Glucose | WT | 1.24 ± 0.12 | 33.92 ± 0.62 | 0.36 ± 0.01 |
|  | EVO5 | 0.93 ± 0.02 | 37.33 ± 1.25 | 0.63 ± 0.01 |
| Glucose + 30 mg L^-1^ PCA | WT | 0.96 ± 0.02 | 37.33 ± 2.87 | 0.55 ± 0.01 |
|  | EVO5 | 0.97 ± 0.01 | 38.00 ± 0.82 | 0.65 ± 0.02 |
| Acetate | WT | 1.90 ± 0.04 | 15.00 ± 0.00 | 0.30 ± 0.02 |
|  | EVO5 | 1.88 ± 0.13 | 7.27 ± 0.41 | 0.23 ± 0.01 |
| L-lactate | WT | 0.90 ± 0.00 | 17.17 ± 0.85 | 0.30 ± 0.00 |
|  | EVO5 | 0.91 ± 0.02 | 4.80 ± 0.28 | 0.16 ± 0.02 |
| Gluconate | WT | 1.00 ± 0.04 | 26.00 ± 0.00 | 0.32 ± 0.12 |
|  | EVO5 | 0.99 ± 0.02 | 24.00 ± 1.41 | 0.36 ± 0.04 |

# References

Buchholz, J., Graf, M., Blombach, B., and Takors, R. (2014). Improving the carbon balance of fermentations by total carbon analyses. *Biochemical engineering journal*, 90, 162-169. doi: 10.1016/j.bej.2014.06.007
